# Suicidal behavior in German military service members: An analysis of attempted- and completed suicides between 2010 and 2016

**DOI:** 10.1371/journal.pone.0256104

**Published:** 2021-08-19

**Authors:** Christian Helms, Florian Wertenauer, Kai-Uwe Spaniol, Peter Lutz Zimmermann, Gerd-Dieter Willmund

**Affiliations:** 1 German Armed Forces Hospital Berlin, Centre for Military Mental Health, Berlin, Germany; 2 Academic Unit of Psychiatry and Addiction Medicine, Canberra Hospital, Australian National University Medical School, Woden, ACT, Australia; 3 6. Psychiatrische Abteilung, Sozialmedizinisches Zentrum Baumgartner Höhe und Otto-Wagner-Spital, Wien, Austria; 4 Bundeswehr Institute of Preventive Medicine, Div. B: Health Information, Andernach, Germany; University of Toronto, CANADA

## Abstract

Studies identified service members of the United States (US) Armed Forces as a high-risk group for suicide. A significant increase in the suicide rate in the US Armed Forces was found in recent years. To date, there is no military suicide statistic available for the German Armed Forces. This study examined attempted and completed suicides in active service members of the German Armed Forces between 2010 and 2016 retrospectively, on the basis of archived personal and medical records in the central archives of the Medical Service of German Armed Forces. The primary goal was to establish a suicide-statistic for the German Armed Forces and to calculate and compare the suicides rates with the German population. Secondary every case’s data was analysed the groups of attempted and completed suicides were compared. 262 attempted suicides and 148 completed suicides were included in this study (N = 410). The suicide rates of the German Armed Forces peaked over the years 2014–2015 with a suicide rate of 15–16/100.000 active military service members and exceeded the civilian suicide rate in Germany of around 12/100.000 people during those years, although no general trend could be determined. These service members were mostly young men (attempted suicide 81.7%, completed suicide 99.3%), at the age of 17 - <35 years old (87% attempted suicide, 68,3% completed suicide), and were employed less than 6 years in the German Armed Forces (attempted suicide 72.9%, completed suicide 46.3%). Service members with attempted suicides belonged mostly to the military North Atlantic Treaty Organization (NATO)-rank-group for other ranks (lowermost military professionals) OR-1 –OR-4 (48.1%) or to the rank-group OR-6 –OR-9 in the group of completed suicides (34.5%). Only in about one third of cases a psychiatric diagnosis could be found in the records. Most frequent diagnoses were neurotic, stress-related and somatoform disorders (International Classification of Diseases Tenth Revision^ICD-10: F4) in 46.8%, and affective disorders (ICD-10: F3) in 43.3% of all cases. In the majority of cases there were signs for potential stressors in the private sector (attempted suicide 90.6%, completed suicide 82.6%). No typical risk factors which would enable a specific prevention could be identified in this analysis. Therefore, should preventive strategies be aiming at a multi-level intervention program.

## Introduction

Suicide is one of the leading causes of death in Germany. From 2010 to 2016 the German suicide statistic showed a relatively stable suicide rate in the general German population of around 12/100.000 [1]. More people died as a result of self-harm than due to car accidents in Germany in 2014 [2]. Especially middle aged men between 40 to 60 years are at risk for suicide [3]. But even though young men don’t show the greatest statistical risk, suicide, next to accidents, is one of the leading causes of death in men between the age of 15–30 years [4].

The aetiology of suicidal behaviour appears to be multifactorial, with genetic-biological factors, such as neuronal receptor mutations or genetic variations, mental disorders and other medical conditions, as well as external socio-economic factors, such as employment status, educational background and living conditions, and internal personality factors, for example emotionally unstable personality disorder traits and lack of functional coping strategies [5]. The interaction of these factors may lead to suicidal crises. However, there is still no valid tool to predict suicidal behaviour for individual patients [6].

Also, in the Bundeswehr (Bw) suicide is one of the leading causes of death and has been reported in 25–30% of the death cases in active service members. Within the armed forces, however, the sensitive military structure, which relies on putting one’s trust and sometimes even one’s life in the capability of others, is damaged. This can have serious negative effects on every soldier of an unit. This disruptive effect can especially be seen, when suicides occur on duty or during a military deployment abroad. Emotions like helplessness and anger, and a lack of understanding can persist in close service members of the deceased for months [7].

Suicidal behaviour of service members was the subject of multiple mostly US-American studies in the past years. One of these studies from 2011 showed that the personnel of the US Armed Forces was at high risk for suicide. There was an increase in the suicide rate (suicides per 100.000 service members) from 10.3 in 2003 up to 15.8 in 2015 [8]. Retrospective analysis showed that the suicide rates doubled between 2004 and 2008. Furthermore, there was a two-fold increase in the number of psychiatric outpatients between 2000 and 2008 in the American military. 50% of the service members who died by suicide had previously been treated for a psychiatric disorder as an outpatient and 17% had been treated on an inpatient psychiatric ward [9].

A connection between the steadily increasing suicide rates since 2004 and the start of missions in Iraq and Afghanistan could be suspected, but studies investigating this relationship delivered inconsistent results [10].

To date, there is no comparable suicide statistic available for the German Armed Forces (GAF). A first epidemiological analysis will be presented in this study by calculating the suicide rates for the German Armed Forces and comparing these rates with the German population in general.

One German study investigated suicidal behaviour in German service members who had been treated as psychiatric inpatients in a hospital of the German Armed Forces in Berlin or Hamburg between 2005 and 2008. Service members with suicidal ideations or with a -attempted suicides in the past were more frequently in their basic military training and significant differences in family status and educational level were found in this analysis. An influence of military missions abroad was not investigated in the study [11].

This study is part of a suicide research program, which was initiated to investigate suicides within the German Armed Forces. Primary goal was to establish a full survey suicide register for military suicides in the GAF, to calculate suicide rates and put those into comparison with the general German population and theraby identify possible specific risk groups. Secondary, the suicidal behaviour of service members in the German Armed Forces were analysed for contributing factors. Therefore, all known attempted and completed suicides of active German Service members between 2010 and 2016 were included in this study. The data were extracted from critical incident reports (CIR) and from archived medical files. The data set included socio-demographic information (e.g. age, gender, military rank, time of employment) and information on potential stress factors (e. g. conflicts with loved ones, job related stress, etc.) that may have accompanied the suicidal crisis. data on medical history and information about military deployments in the past. For analysis the data set was divided in attempted suicides and completed suicides, to test for significant differences between those incidents and common aspect.

## Methods

The data extract of the study data was carried out exclusively by medical personnel within their official activities in accordance with the current data protection laws (DSGVO) of the Federal Republic of Germany. The anonymized data was generally compiled for official reporting purposes. The transfer of data to the study group involved in the study was exclusively anonymized, sorted by year.

Special personal data (such as name, address, date of birth, location of the event) was not provided in the data extract. It was not possible to assign place of residence or even place of employment. Nevertheless, it was not possible to trace back to individuals.

This study was officially registered at the German Armed Forces Data Protection Authority and meets national ethics and privacy requirements. The study is approved by German Federal Ministry of Defense and the Medical Service Academy, Munich. Medical ethical approval was sought before analysis, but the ethics board ruled that observational studies using anonymized data are not subject to ethical approval.

All death reports (CIR) of active soldiers between 2010 and 2016 were scanned for signs for a suicidal intention by a medical doctor (general practitioner) in the Institute for Military Medicine Statistic und Reporting” (WehrMedStatInstBw) in Andernach. In cooperation with the German Prosecution autopsy reports were evaluated to identify all possible suicides. These potential suicide cases were re-evaluated by an independent specialized psychiatrist. All thereby extracted cases were re-analysed by an independent psychiatrist from the Centre for Military Mental Health in Berlin, before being included in this study. A case was defined as suicide, if there were since for suicidal thoughts (e. g. letter of goodbye) and the autopsy reports showed signs for s deliberate self harm. This approach was chosen to minimize the dark figure.

In addition to that, all critical incident reports regarding attempted suicides of active service members in the German Armed Forces between 2010 and 2016 were included in this retrospective analysis. Attempted suicide was defined as attempts or incidences of self harm with the intention or ideation to die as a result.

The existing data were analysed for every included study case. First, a short medical report (S-Blatt; “report of suicidal behaviour”) containing the affected soldier’s medical information provided by the treating GP, which was available for most cases. This document included socio demographic information, relationship status, children, distance between residence and workplace, past attempted suicides and medical diagnoses. Furthermore, the past military medical history was available for former (incl. deceased) service members. Those files contain documents of all medical examinations or procedures, which took place during the military employment.

The data was archived in Microsoft Excel 2008 and the statistical analyses were performed with SPSS 23rd Version. Differences between the nominal and ordinal items of the two groups were examined with Pearson’s chi-square test. The significance level was defined with α = 5% (p-value < 0.05).

In cooperation with the press information centre of the German Armed Forces Medical Command we were able to analyse the suicide data in relation to mean employment figures for each year. These figures were documented every December and included all active employed service members at that time. The military suicide rates per 100.000 service members were calculated and compared with the civilian suicide rates for each year. These civilian rates were calculated using public data from “Statista”, an internet-platform of a German statistic institute (population of Germany and suicides per year in Germany).

The medical files were examined to extract major psychiatric diagnoses (ICD-10-Code group F, International Classification of Diseases, 10^th^ Version), treatment settings, other severe health problems, previous consultation with military psychiatrist (yes/no) and consultation with a military general practitioner within the last month (yes/no).

The data were screened for signs of conflicts or problems (in both the patient’s private or work life) at the time of the suicide attempt. Three subcategories were formed to describe conflicts in the private life. These were “separation from a partner”, “conflicts with a partner” and “conflicts with the parents”. Workplace difficulties were grouped in “conflicts with other service members” and “conflicts with superiors”. General problems with the military structure, the end of duty within the next 3 months (except conscripts) and current basic training were evaluated separately.

Past military missions abroad and deployment related problems were recorded in a separate set of items. Dichotomous items contained “any military mission abroad”, “more than one mission abroad”, “trauma during deployment”, “relationship difficulties during deployment”, “repatriations of any kind” and “suicidal crisis during a military mission”. After returning from deployment every soldier received a medical check-up. Signs of any stress related mental disorder were recorded in this examination as a separate item. Sometimes the “Post-Traumatic Stress Scale 10” (PTSS-10) was filled out by the regarding soldier.

Via self-assessment questionnaires service members are asked to give information about their socio-demographic and medical background on several occasions during their employment at the German Armed Forces. A number of factors that may impact on suicidality were extracted from those questionnaires. Next to information about schooling, job training and work before entering the military service, data about the current relationship status and biological children were documented. Furthermore, service members are asked about their medical family history including mental disorders and alcoholism, previous conflicts with the law, drug abuse and financial debt.

The medical reports were scanned for past suicide attempts, information of a broken home situation while growing up, the relationship status of the parents, and a dead or severely sick relative.

Finally, the attempted or completed suicide was analysed itself. The chosen method of self-harm was recorded and compared between the two sub-groups (attempted and completed suicide). Information on the influence of alcohol, the location of the attempted suicide, and the clothes worn during the self-harm was extracted from the files.

When no information could be extracted from the files, the item remained blank. These items were treated as missing data and were not be integrated in the analysis.

A binary logistic regression model was calculated to estimate the potential predictive value of each significant item. The likelihood-ratio-regression in the stepwise forward method was used for the selected items. Only objectifiable items, such as personal data, medical diagnoses, private and professional conflicts, relationship status and abroad mission information were included in the analysis to ensure the validity of the information.

## Results

In total, 410 cases were included in this study. 262 service members (63.9%) had committed an attempted suicide and 148 cases (36.1%) were completed suicides. In the following, to the group of attempted suicides will be referred to as “attempted suicide”-group and to the group of completed suicides as “suicide”-group.

The number of male service members was significantly higher in both the attempted suicides- and the suicide group. There was only one female soldier who died by suicide in the study period. The personal information of the included cases is shown in Table 1.

**Table 1:** Socio-demographic information.

	Attempted suicide (%)	Completed suicide (%)	p-value
N = 410	262 (63.9)	148 (36.1)	
Gender	214 (81.7)/	147 (99.3)/	<0.001
(male/female)	48 (18.3)	1 (0.7)	
Employment status:			<0.001
Conscript (GWDL)	20 (7.6)	5 (3.4)	
Regulars (SaZ)	190 (72.5)	92 (62.2)	
Professionals (BS)	35 (13.4)	46 (31.1)	
Voluntary conscript (FWDL)	17 (6.5)	3 (2.0)	
Others	0 (0.0)	1 (0.7)	
Military rank:			<0.001
Men (OR-1 –OR-4)	126 (48.1)	45 (30.4)	
Corporal (OR-5)	50 (19.1)	26 (17.6)	
Sergeant (OR-6 –OR-9)	66 (25.2)	51 (34.5)	
Officer (OF)	20 (7.6)	26 (17.6)	
Age band:			<0.001
16 - <25 years	123 (46.9)	43 (29.1)	
25 - <35 years	105 (40.1)	58 (39.2)	
35 - <45 years	18 (6.9)	16 (10.8)	
45 - <55 years	16 (6.1)	27 (18.2)	
55 - <65 years	0 (0.0)	4 (2.7)	
Time of service band:			<0.001
1–6 years	191 (72.9)	68 (46.3)	
7–12 years	36 (13.7)	32 (21.8)	
13–20 years	18 (6.9)	10 (6.8)	
>20 years	17 (6.5)	37 (25.2)	
Graduation: middle school or higher	95 (63.3)	112 (79.5)	0.007
Apprenticeship: graduated	75 (51.7)	82 (55.8)	0.001
Work: employed occupation learned	50 (36.2)	75 (52.4)	0.042
Parents: early separation	35 (36.1)	20 (37.7)	0.014
Broken home	36 (37.9)	26 (31.3)	0.359
Death/ severe sickness of a close family member	23 (20.0)	24 (20.5)	0.923
Current relationship	76 (35.5)	50 (43.1)	0.175
Children	30 (17.4)	39 (27.9)	0.027

The results show that suicidal service members (both attempted and completed suicides) were mostly regulars, aged 16–35 years, had the military NATO-rank of OR-1 to OR-4 or OR-6 to OR-9 and were employed by the German Armed Forces for less than 6 years.

The suicide group showed a bivalve age distribution. After to the younger male service members (68.3% in the group of <35 years), over 18% of all cases occurred in the group of the 45 –<55-year-old service members.

In the completed suicide group, a significantly higher number of service members had received middle or higher school education (79.5% vs. 63.3%; p = 0.007), finished at least one job training (55.8% vs. 51.7%; p = 0.001) and grew up with separated parents (37.7% vs. 36.1%; p = 0.014).

Suicidal soldiers were more likely single at the time of the suicidal crisis (64.5% attempted suicide group vs. 56.9% completed suicide group; p = 0.175).

27.9% of the deceased service members had children on their own at the time they committed suicide, which was significantly higher than in the suicide attempt group (17.4%; p = 0.027).

In the general population in Germany (GER Population), the suicide rate appeared to be stable between 2010 and 2015 at about 12 to 13 per 100.000 population. These rates appear to be comparable to other European neighbouring countries.

**Fig 1:**
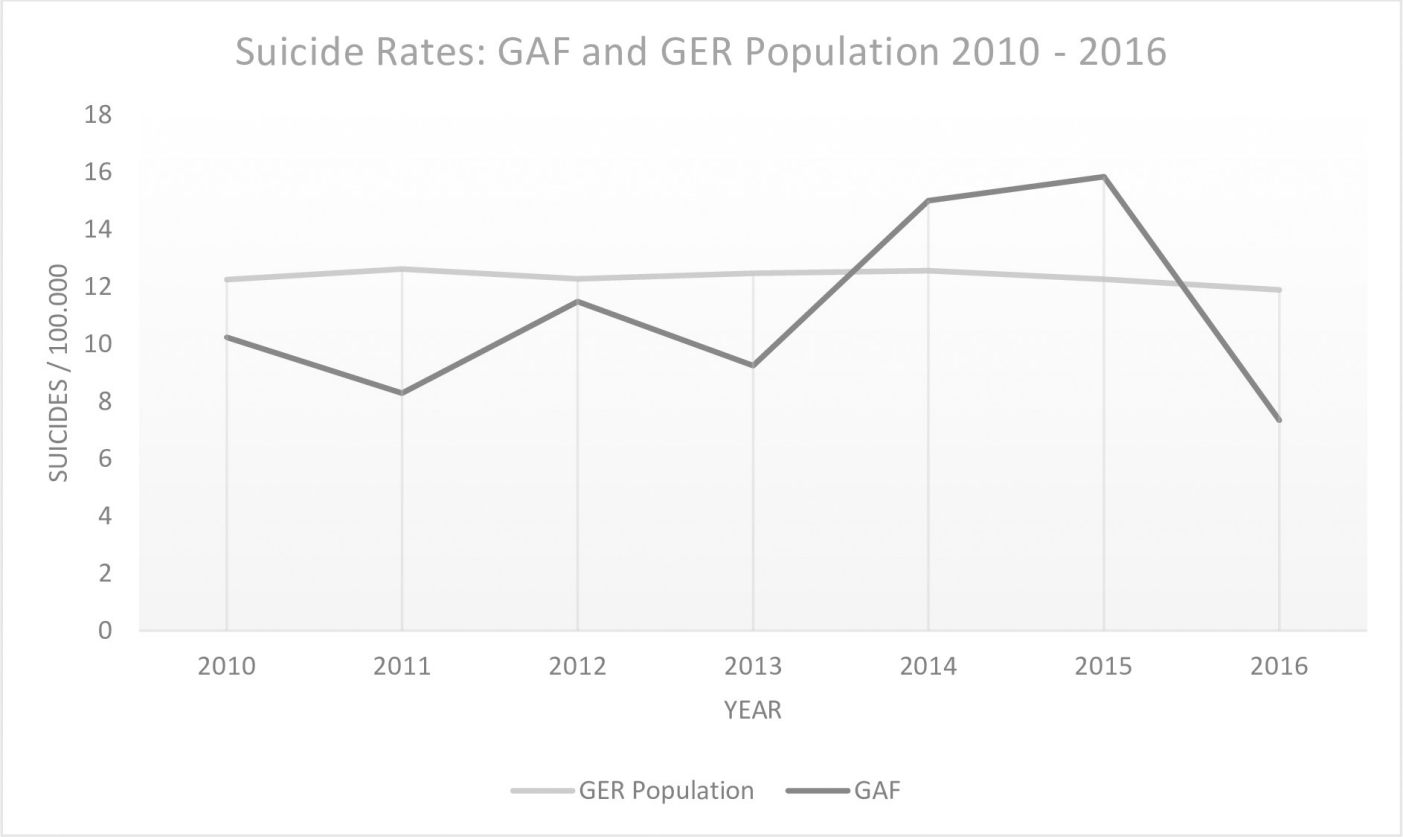
Suicide rates per 100.000 individuals of German Armed Forces/Bundeswehr (GAF) and German population (GER population) 2010–2016.

The suicide rates in the German Armed Forces appeared to be lower than in the general population over the years 2010–2013 with a rate of 8–10/100.000 service members. This rate increased in 2014 and 2015 to a suicide rate of 15–16/100.000 service members and exceeded the civilian suicide rate in Germany (see Fig 1).

About one third of service members of both groups received psychotherapeutic treatment during their service (attempted suicide 32.0%, completed suicide 30.3%; p = 0.523). ICD-group F3 affective disorders (attempted suicide 43.1%, completed suicide 43.5%) and ICD-group F4 “neurotic, stress-related and somatoform disorders” (attempted suicide 45.4%, completed suicide 48.2%) were the most frequent diagnoses in the medical records. The diagnosis PTSD was assigned in about 4–6% of all cases in both groups. Physical health problems could rarely be found in the files, but the incidence was significantly higher in staff who died as a result of an attempted suicide (9.5%; p = 0.001). Service members of both groups showed a family history of mental health disorders in about 28% and of alcohol dependency disorders (ADD) in about 24–36% of the cases (no statistically significant differences p = 0.109). About one third of cases had one or more documented encounters with a psychiatrist (p = 0.644). There was a significantly higher rate of encounters with the military GP within the month prior to the incident in the suicide attempt group (68.3%; p <0.001). The reason for the encounter was not specified in this analysis (see Table 2).

**Table 2:** Medical information.

	Suicide attempt (%)	Suicide (%)	p-value
ICD-10 group:			0.851
F3	56 (43.1)	37 (43.5)	
F4	59 (45.4)	41 (48.2)	
PTSD	13 (5.5)	4 (4.3)	0.650
Psychotherapeutic treatment:			0.523
None	153 (68.0)	92 (69.7)	
Outpatient/	41 (18.2)/	27 (20.5)/	
Inpatient	31 (13.8)	13 (9.8)	
Severe health problems	5 (2.0)	12 (9.5)	0.001
Mental disorders in the family	24 (27.9)	24 (27.6)	0.962
Alcohol dependency problems in family	24 (35.8)	27 (24.1)	0.109
Contact to military GP (within the last month)	82 (68.3)	52 (38.2)	<0.001
Psychiatrist consultation	45 (31.5)	49 (34.0)	0.644

In most cases in both groups signs of interpersonal conflicts in their private life (suicide attempt group 90.6% vs. suicide group 82.6%; p = 0.032). A significantly higher percentage of cases in the suicide group recently separated from their partner (37.6% vs. 24.3%; p = 0.024). The rates for relationship-conflicts in general did not differ significantly (p = 0.120).

Within the suicide attempt group, there were significantly more duty related conflicts (37.5% vs. 22.7%; p 0.006) and significantly more problems with the military structure (20.8% vs. 13.7%; p <0.001), compared to the suicide group. Conflicts with the military superior were found in about 15% of the cases in both groups (p = 0.868), but conflicts with other service members were significantly more often in the attempted suicide group (17.8% vs. 7.7%; p = 0.018).

A significantly larger number of service members in the suicide group were about to leave the German Forces within the next months (25.9% vs. 3.9%; P <0.001). No significant group differences could be found for the item “during basic training” (p = 0.166). Financial debts were significantly more frequent in the group of attempted suicide (19.5% vs. 9.5%; p = 0.038). The analysis of the other items revealed no statistically significant differences.

There were no significant differences between the two groups regarding their “criminal record before joining the forces”, drug consumption or disciplinary penalties. For detailed information on the potential conflicts see Table 3.

**Table 3:** Potential conflicts and stressors.

	Attempted suicide (%)	Completed suicide (%)	p-value
Private conflicts:	213 (90.6)	90 (82.6)	0.032
Separation from partner	36 (24.3)	38 (37.6)	0.024
Conflicts with partner	85 (56.3)	68 (66.0)	0.120
Conflicts with parents	31 (23.0)	18 (18.2)	0.375
Official conflicts:	81 (37.5)	27 (22.7)	0.006
Conflicts with service members	24 (17.8)	9 (7.7)	0.018
Conflicts with superiors	19 (14.5)	18 (15.3)	0.868
Disciplinary penalties	26 (12.3)	16 (11.1)	0.741
Problems with the military structure	48 (20.8)	18 (13.7)	<0.001
End of duty	10 (3.9)	15 (25.9)	<0.001
During basic training	15 (5.7)	4 (2.7)	0.166
Criminal record before joining the forces	5 (2.6)	5 (3.5)	0.623
Drug abuse	24 (19.8)	16 (11.3)	0.057
Financial debt	22 (19.5)	10 (9.5)	0.038

The analysis of the deployment history revealed that service members of the suicide group had been deployed significantly more frequently to one or more military missions abroad (32.2% vs. 14.0%; p <0.001 one mission, 12.5% vs. 4.6%; p <0.001 more than one mission). A potential psychological trauma during mission occurred in 2,7% of the cases in the suicide group and in 3,1% of the cases in the attempted suicide group (p <0.001). Psychological traumatisation, repatriations (medical evacuation) as well as suicidal crises during an abroad mission occurred in a negligible number of cases (< 5%), so that the statistical analysis had no clinical relevance. Table 4 summarizes the data about abroad missions in the past.

**Table 4:** Deployment information.

	Attempted suicide (%)	Completed Suicide (%)	p-value
Abroad missions in the past	29 (14.0)	47 (32.2)	<0.001
More than 1 mission	9 (4.6)	18 (12.5)	<0.001
Traumatization during mission	6 (3.1)	4 (2.7)	<0.001
Suicide during abroad mission	3 (1.1)	3 (2.1)	0.464
Repatriation	8 (4.0)	5 (3.5)	0.713
Symptoms in returnee check-up	2 (1.1)	11 (8.8)	<0.001

The different methods used in the suicide attempts are listed in Table 5. There were significant differences between the two study groups (all p-values <0.001). Service members within the suicide attempt group overdosed on medication in 38.2% (vs. 6.1% of the suicide group) and deliberately cut themselves along the wrists in 31.3% (2.0% of the suicide group). Service members of the suicide group mostly used strangulation via self-hanging (35.1% vs. 6.5% in the attempted suicide group), firearms (12.8% vs. 1.1% in the attempted suicide group) or caused a deliberately train accident (12.8% vs. 0.4% in the attempted suicide group). Attempted suicides were significantly more often conducted under the influence of alcohol (26.9% vs. 11.4%, p = 0.001).

**Table 5:** Suicide methods and accompanying incident factors.

	Suicide attempt (%)	Suicide (%)	p-value
**Type of self-harm:**			<0.001
Medication	100 (38.2)	9 (6.1)	
Illicit drugs	1 (0.4)	2 (1.4)	
Cuts along the wrists	82 (31.3)	3 (2.0)	
Other cuts	12 (4.6)	3 (2.0)	
Self-hanging	17 (6.5)	52 (35.1)	
Fall or jump	10 (3.8)	7 (4.7)	
Provoked traffic accident	11 (4.2)	8 (5.4)	
Shooting	3 (1.1)	19 (12.8)	
CO-intoxication	12 (4.6)	10 (6.8)	
Suffocation / drowning	2 (0.8)	7 (4.7)	
Train accidents	1 (0.4)	19 (12.8)	
Other	10 (3.8)	9 (6.1)	
Incident factors:			
Military facility	58 (22.2)	31 (20.9)	0.764
Military uniform	26 (10.0)	27 (18.4)	0.015
Influenced by alcohol	66 (26.9)	14 (11.4)	0.001
Suicide attempts in the past	32 (13.4)	15 (12.6)	0.825
Suicide attempts in the family	4 (4.2)	4 (4.9)	0.806
Suicide attempts among acquaintances	2 (2.3)	3 (4.8)	0.415

In both groups the self-harm was mostly performed in civilian clothes and outside of military facilities. In up to 18% of the cases a military uniform had been worn and up to 22% of the suicide attempts were executed within a military facility. There were no significant differences between the two groups.

The socio-demographic and medical items and those used to describe the potential conflict situation at the time of the suicidal crisis were included in a binary logistic regression analysis. 40 suicide cases and 63 suicide attempt cases were included in the stepwise backward binary logistic likelihood-ratio-regression analysis (summary in Table 6). Other cases could not be integrated in this analysis due to missing data.

**Table 6:** Likelihood-ratio-regression analysis to distinguish between attempted and completed suicides.

Item	Wald	Sig.
Age group	10.97	< 0.001
Professional conflicts	6.34	0.005
Private conflicts	4.01	0.045
Separation from partner	2.87	0.09
Conflicts with superiors	4.39	0.036
Missions abroad	2.96	0.085
School graduation	3.043	0.081

The age group showed the strongest significant influence in differentiating the two groups (p < 0.001). Furthermore, the accompanying conflict constellation was integrated in the regression analysis with a significant p-value (professional conflicts p = 0.005, private conflicts p = 0.045). Separation from a partner (p = 0.09), conflicts with military superiors (p = 0.036) as well as a participation in at least one deployment in an abroad mission (p = 0.085) and schooling (p = 0.081) remained important factors to discriminate those two clinical groups. In this regression model the–log likelihood value reached its highest values with 89.90 and a predictive power with Nagelkerke’s R^2^ = 0.50. Using the regression model 52 suicide attempt cases (82.5%) and 28 suicide cases (70.0%) could be identified correctly. In total the correctly predicted percentage is 77.7%.

## Discussion

In this study, all documented cases of active service members in the GAF who committed a suicidal incident (attempted and completed) between 2010 and 2016 were analysed retrospectively on the basis of different files.

The suicide rates for the German Armed Forces over the years 2010–2016 were calculated in relation to the general personnel figures for each year. Compared to the non-weighted civilian suicide rates in Germany over the years 2010–2016, the military suicide rates showed an increase in the suicide rates over the years 2013–2014 to rates of 15–16/100.000 service members and even exceeded the civilian suicide figures in these two years (12–13/100.000 people). However, due to the rather high fluctuations in the military suicide figures, no general trend could be determined in our analysis. The suicide rate for the German Armed Forces in 2016 declines to 7/100.000 service members, which is lower than the civilian suicide rate in Germany in 2016 of 11.9/100.000 people).

Suicidality seemed to occur mostly in young male service members. More than 80% of all suicidal incidents (fatal and non-fatal) were committed by service members in the age between 16 - <35 years. 88% of all included cases were men. Most of these military service members had a low military rank (more than 60% OR-1 –OR-5), were employed in the German Armed Forces for less than 4 years, and served as regulars.

But it has to be mentioned, that no gender- and age-controlled suicides rates were calculated in this study. Therefore, it remains unclear, if young male military service members have a greater suicide risk than others. In a further study, it is planned to analyse every age group and military rank group in order to investigate whether there are any high-risk groups for suicidal behaviour within the military structure.

In addition to that, due to the fact that suicides occur up to three times more frequently in men than in women [12], further studies should also calculate gender specific suicide rates and compare gender specific rates of the general population with the military suicide rates of male and female service members.

The suicide group who performed showed a u-shaped age distribution. 18% of all cases occurred in the group of the 45 –<55-year-old service members. It is likely, that because of a smaller total personnel figure in this older age group (45 -<55 years), this relatively high total suicide count could lead to the highest suicide rate of all age groups of the German Armed Forces.

The age group of 45 - <55-year-old service members consists mostly experienced service members with a higher military rank (e.g. officers) who often served in the German Armed Forces for many years mostly without significant problems. A Canadian study suggested in their study on military service members during and after peace-keeping missions, that a military lifestyle could lead to interpersonal problems, unhealthy alcohol consumption and other burdens. It could be suggested that the incidence of mental disorders increases with age and that older service members are more likely to develop such disorders [13]. There are no Germany studies regarding an increasing risk for mental disorders in older service members available, so further research is warranted.

In the group comparison, the regression model revealed a strong influence of age to differentiate the attempted suicide group from the suicide group, with older service members appearing to be at higher risk of completed suicides.

Deployment on a military mission abroad was identified in this analysis as another factor suited to discriminate the two groups investigated in this study. Which could be explained by the fact, that more experienced service members, are more likely to have history of military missions abroad. So, this item could be a well-suited indicator on how experienced a soldier is in the military field.

On the other hand, items such as private and professional conflict appeared to be important, non-confounding items, especially for service members in the attempted suicide group. Additionally, school education was a significant factor in the regression model. Where a lower school education could be seen more frequent in the attempted suicide group.

However, despite the significant differences between the two groups, many similarities could be found, such as the incidence of private conflicts, suicide attempts in the past, a mental disorder in the family history, a broken home situation or psychotherapeutic treatment during the time of service.

Compared to the German suicide study in the German Armed Forces from 2012 by Zimmermann et al. [11], the present study identified similar characteristics of service members in a suicidal crisis. In an ad on study, the Centre for Military Mental Health (PTZ) in Berlin compared this study cohort with a healthy control group in order to better understand the risk factors for suicidal crises in service members [14].

The analysis of the different methods of self-harm revealed remarkable differences between the two groups. Attempted suicides were most frequently conducted by medication overdoses or cuts along the wrists or arms. About a quarter of this group were under the influence of alcohol. On the other hand, completed suicides were most frequently a result of self-hanging, fatal shooting, or deliberate train accidents.

In the general German population only 4.4% of all suicides were conducted by firearms [4]. Many studies before have shown that the access to lethal means is a risk factor for suicide [15]. A similar process can be suspected in light of this data, where the use of firearms was the second most frequent method of completed suicides. Even though the German Armed Forces have strict regulations regarding firearms, where service members are only allowed to carry firearms during guard duty or on the shooting range, a potential risk must be assumed. At the same time, not only military weapons should be considered in this regard. Even though, the strict gun regulations in Germany severely limit the possibility of gun ownership, many German service members acquire hunting licences and thereby the licence to buy and carry own weapons. No data about existing hunting licences was available for this study.

Only a low rate of attempted suicides used the three mentioned lethal means (self-hanging, firearms and train accident) of the suicide group. In the past those suicide attempts were seen as “failed completed suicide attempts” [16]. When analyses were restricted to these three methods only, no significant differences to the results could be found regarding the socio-demographic data, such as age, military status, rank group or time of employment.

One possible explanation is, that the phaenomena of attempted and completed suicides are not the same after all. Maybe it could be seen as two different degrees of the same continuum. Where attempted suicides may still have a non-verbal help seeking aspect or could be seen as a non-functional coping strategy to seek professional help, in completed suicides the intention to die by the self-harm is striking. But, the deduction, that attempted suicides are not serious signs for a crisis, would be wrong. All attempted suicides reflect a patient in need for help in their specific situation. It could rather help in our clinical practice to take the chosen lethal mean into account about our recommended follow up procedure, by knowing, that some methods could be seen as red flags for the patients’ intention to die.

Taking those findings into account it seems important to think about preventive strategies that are targeting the special needs of young male service members as well as older well experienced service members. In US men do less often get diagnosed with depressive symptoms, but international studies found evidence that “many more men” could suffer from depressive symptoms [17]. It rather seems like men do not seek professional help as often as women. Also, a retrospective study in the GAF regarding mental health problems after missions abroad showed, that a rate of only 10–20% of the service members with manifest psychiatric symptoms sought professional help within the military mental health services.

It was noteworthy that a rate of less than on third of all service members in this study had been in psychiatric treatment. It can be assumed, however, as shown in other studies in the general population before, that in about 90% of suicide cases a psychiatric disorder is present at the time of the suicide [18]. By optimizing the early recognition of mental health problems in the military personal there might be a great potential to reduce the suicide rate in the German Armed Forces by optimising the early recognition and treatment of psychiatric disorders.

Maybe this phaenomenon could be seen from the background of an established male gender role in the western society. Especially in young men, those gender roles seem to be important guidelines, that help to separate from the family and to identify as an independent person. “Masculinity appeared to require being strong and silent about emotions” [19]. Especially in the military setting those masculinity rules could play an important part in the behaviour of young men.

Following this concept, the ways of perceiving depressive symptoms might be comparable in men and women, but the ways of expressing the symptoms and dealing with the situation seem to be quite different. The gender role theory distinguishes a feminine coping style (emotion focused) from a masculine coping style (problem focused) in dealing with emotional stressors [20]. Brownhill et al., 2005 established a theory of “masculine enactment of emotional distress” which starts with avoidance (“overwork”), followed by numbing (alcohol, self-medication”) and lead to escaping behaviours that may escalate to violence and suicide. The mentioned gender-related differences seem to influence the expression of depression not the experience of the depression [21].

It could be recommended to create more awareness of those “male coping” strategies, such as overwork, alcohol consumption, and escape behaviours, in the military context, to offer low threshold problem focused help-options.

In addition to that a fear of negative consequences of someone’s carrier and a fear of stigmatisation seem to be a quite common barrier in service members of the GAF. Especially the fact, that the available therapeutic offers and medical help runs by the GAF, seems to make it even harder for service members to seek help. Therefore, comes the seek for help in case of a mental disorder always along with a disclosure towards the GAF. Service members also described negative stereotypes towards mental disorders such as being weak, incompetence, self-blame, or malingering [22]. A German study of service members who served in ISAF between 2009–2010 should that only half of the staff thought professional help in case of mental disorders. One of the main points in optimizing the medical and psychotherapeutic treatment should be therefore aiming towards stigma reduction and by that to a better help-seeking culture in the GAF.

## Supporting information

S1 File (XLSX)

## References

[1] Fiedler G. Nationales Suizidpräventionsprogramm für Deutschland Suizide in Deutschland 2013. Available from: www.naspro.de/ Suizidzahlen2013.pdf [cited 2021 Aug 10]

[2] Statista: Das Statistik-Portal. Anzahl der Getöteten bei Straßenverkehrsunfällen in Deutschland von 1950 bis 2020. Available from: https://de.statista.com/statistik/daten/studie/185/umfrage/todesfaelle-im-strassenverkehr/#professional [cited 2021 Aug 10]

[3] Hem E, Haldorsen T, Aasland OG, et al. Suicide rates according to education with a particular focus on physicians in Norway 1960–2000. *Psychol Med* 2005; 35: 873–880. doi: 10.1017/s0033291704003344 15997607

[4] Statistisches Bundesamt. Gestorbene: Deutschland, Jahre, Todesursachen, Geschlecht, Altersgruppen (2017). Todesursachenstatistik, Statistisches Bundesamt (Destatis). Available from: https://www.destatis.de/DE/Themen/Querschnitt/Jahrbuch/jb-gesundheit.pdf?__blob=publicationFile [cited 2021 Aug 10]

[5] Fenner D. Welches sind die Gründe für eine Selbsttötung? Kritische Analyse verschiedener Suizidtypen. E-Journal Philosophie der Psychologie 2006; 6. Available from: www.phps.at/texte/FennerD1.pdf [cited 2021 Aug 10]

[6] Fragala MR, McCaughey BG. Suicide following Medical/Physical Evaluation Boards: a complication unique to military psychiatry. *Mil Med* 1991; 156: 206–209. 2030846

[7] Willmund G-D, Helms C, Spaniol K-U, et al. Suizidalität in Streitkräften–Risikofaktoren für vollendete Selbsttötungen von Soldaten. *Wehrmed*. *Mschr*. *Heft 1/2016*, January 2016, pp. 15–18.

[8] Ramchand R, Acosta J, Burns RM, et al. *The War Within—Preventing Suicide in the U*.*S*. *Army*. RAND, 2011. 10.7249/rhq-01-01-02 PMC4945209 28083158

[9] Bachynski KE, Canham-Chervak M, Black SA, et al. Mental health risk factors for suicides in the US Army, 2007–8. *Inj Prev* 2012; 18: 405–412. doi: 10.1136/injuryprev-2011-040112 22398362

[10] Kuehn BM. Soldier Suicide Rates Continue to Rise. *JAMA* 2009; 301: 1111. doi: 10.1001/jama.2009.342 19293405

[11] Zimmermann P, Höllmer H, Guhn A, et al. Prädiktoren suizidalen Verhaltens bei Bundeswehrsoldaten. *Nervenarzt* 2012; 83: 359–365. doi: 10.1007/s00115-010-3243-x 21424415

[12] Thomas CS, Read DA, Mellsop GW. New Zealand suicides 1984–8. *N Z Med J* 1992; 105: 231–233. 1620496

[13] Wong A, Escobar M, Lesage A, et al. Are UN peacekeepers at risk for suicide? *Suicide Life Threat Behav* 2001; 31: 103–112. doi: 10.1521/suli.31.1.103.21305 11326764

[14] Willmund G-D, Heß J, Helms C, et al. Suicides between 2010 and 2014 in the German Armed Forces-Comparison of Suicide Registry Data and a German Armed Forces Survey. *Suicide Life Threat Behav*. Epub ahead of print 17 December 2018. doi: 10.1111/sltb.12534 30556592

[15] Humeau M, Papet N, Jaafari N, et al. Disponibilité des armes à feu et risque suicidaire: revue de la littérature. *Ann Méd-Psychol Rev Psychiatr* 2007; 165: 269–275.

[16] Kriebel J. Suizidprobeme bei jungen Erwachsenen. *Wehrmed*. *Mschr*. *Heft 8/1987*, August 1987, pp. 332–338.

[17] Branney P, White A. Big boys don’t cry: depression and men. *Adv Psychiatr Treat* 2008; 14: 256–262.

[18] Tondo L, Baldessarini RJ. Can Suicide be prevented? *Psychiatric Times*, 2011.

[19] O’Brien R, Hunt K, Hart G. ‘It’s caveman stuff, but that is to a certain extent how guys still operate’: men’s accounts of masculinity and help seeking. *Soc Sci Med* 2005; 61: 503–516. doi: 10.1016/j.socscimed.2004.12.008 15899311

[20] Li CE, DiGiuseppe R, Froh J. The roles of sex, gender, and coping in adolescent depression. *Adolescence* 2006; 41: 409–415. 17225659

[21] Brownhill S, Wilhelm K, Barclay L, et al. ‘Big Build’: Hidden Depression in Men. *Aust N Z J Psychiatry* 2005; 39: 921–931. doi: 10.1080/j.1440-1614.2005.01665.x 16168020

[22] Rüsch N, Rose C, Holzhausen F, et al. Attitudes towards disclosing a mental illness among German soldiers and their comrades. *Psychiatry Res* 2017; 258: 200–206. doi: 10.1016/j.psychres.2017.08.028 28864120

